# Prevalence and associated factors of female sexual dysfunction among primary care practitioners in Malaysia: A cross-sectional study

**DOI:** 10.51866/oa.883

**Published:** 2025-07-29

**Authors:** Rosli Salwa, Abd Halim Haizlene, Md. Yasin Mazapuspavina, Leny Suzana Suddin

**Affiliations:** 1 MBBChBAO, BMedSc, MMed (Fam Med), Department of Primary Care Medicine, Faculty of Medicine, Universiti Teknologi Mara (UiTM), Jalan Hospital, Sungai Buloh, Selangor Malaysia.; 2 Hospital Al-Sultan Abdullah (HASA), Universiti Teknologi MARA (UiTM), Puncak Alam, Selangor, Malaysia. Email: haizlene@uitm.edu.my; 3 MD, Department of Primary Care Medicine, Faculty of Medicine Universiti Teknologi Mara (UiTM), Jalan Hospital, Sungai Buloh, Selangor, Malaysia.; 4 MBBS, MMed (Fam Med), Department of Primary Care Medicine, Faculty of Medicine, Universiti Teknologi Mara (UiTM) Jalan Hospital, Sungai Buloh, Selangor, Malaysia.; 5 Hospital Al-Sultan Abdullah (HASA), Universiti Teknologi MARA (UiTM), Puncak Alam, Selangor, Malaysia; 6 MD, DRPH, Department of Public Health Medicine, Faculty of Medicine Universiti Teknologi Mara (UiTM) Jalan Hospital, Sungai Buloh, Selangor, Malaysia.

**Keywords:** Female sexual dysfunction, Malay version of the female sexual function index, Primary care practitioners

## Abstract

**Introduction:**

Sexual health is integral to quality of life, and female sexual dysfunction (FSD) is a significant public health concern recognised by the World Health Organization. Its prevalence is rising globally, with limited data among primary care practitioners (PCPs). Multifactorial issues such as high work burden in PCPs, coupled with low help-seeking behaviours, contribute to the likelihood of FSD and poor quality of life. This study aimed to determine the prevalence and associated factors of FSD among PCPs in Malaysia.

**Methods:**

This cross-sectional study was conducted using a self-administered online questionnaire for 6 months duration in Malaysia. Sociodemographic, marital and clinical data were collected, and two validated questionnaires namely the Malay Version of the Female Sexual Function Index (MVFSFI) and the Depression, Anxiety and Stress Scale (DASS-21) were utilised. Data analysis included descriptive statistics and logistic regression.

**Results:**

The study included 382 participants. The prevalence of FSD was 14.7% (95% confidence interval [CI] : 11.1, 18.2) among the participants, and the most affected domain was desire disorder (25.4%). Significant associations were observed between sexual activity two or fewer times per month (adjusted odds ratio [aOR]: 4.880; 95% CI: 2.522, 9.442; P<0.001), depression (aOR: 3.450; 95% CI: 1.653, 7.204; P<0.001) and degree-level education (aOR: 2.659; 95% CI: 1.338, 5.285; P=0.005) and FSD.

**Conclusion:**

This study identifies FSD as a prevalent issue among Malaysian PCPs, emphasising its biopsychosocial complex and the need for a holistic approach. Understanding the cultural determinants of FSD is important to tailor screening and intervention strategies.

## Introduction

Sexual health is an important aspect of quality of life and marriage, and sexual dysfunction is defined as a diverse group of disorders characterised by a person’s inability to respond to or experience sexual pleasure.^1^ Female sexual dysfunction (FSD) can be classified into three major domains: female sexual interest/arousal disorder, genito-urinary penetration disorder, and female orgasm disorder. Diagnosis requires symptoms to persist for at least 6 months and cause significant distress while not attributable to other organic conditions or stressors.^2^ Despite being a complex and growing health problem identified as an important public health concern by the World Health Organization,^1,3^ FSD receives less attention compared to male sexual health issues.^4,5^ Nonetheless, addressing FSD is equally significant as part of achieving the Sustainable Development Goals 2030, specifically goal 3 (good health and well-being) and goal 5 (gender equality), emphasising the need to address and empower women’s sexual health.^6^

Recent studies have indicated a growing prevalence of FSD worldwide. An international systematic review showed an increase in FSD prevalence among premenopausal sexually active women, escalating from 40.9% to 50.7% between 2016 and 2020.^7,8^ In Malaysia, the prevalence rose from 29.6% to 68.8% between 2007 and 2020.^9,10^ The prevalence of FSD among medical practitioners was 40% in Tunisia in 2019 and 49.7% in China in 2020,^11,12^ while a study in Malaysia reported a prevalence of 5.5% among healthcare workers (including medical practitioners and allied healthcare workers) in 2013.^13^ Among primary care practitioners (PCPs), the prevalence of FSD is unknown.

PCPs are often the first points of contact in healthcare, and the overall PCP-to-population ratio is estimated to be 2.89 per 10,000 population within the Malaysian primary healthcare system.^14^ PCPs experience issues with work burden, and a study found that 37.1% of public PCPs felt no job satisfaction.^15^ Nelson and Henry revealed that 32.1% of PCPs experienced decreased sexual drive; 19.3% were too tired for sex; and 21.1% did not have time for sex.^16^ Awareness of FSD was reported as only 12% among medical practitioners (including 23 PCPs).^17^ Help-seeking behaviour for FSD in medical practitioners was noted in only 3.15%, which is substantially low compared to the general population, although 82.5% of them reported they needed professional guidance.^12^ Thus, this highlights the need to improve the sexual health and overall well-being of PCPs. Occupational stress has an added effect on female medical practitioners’ sexual dysfunction and quality of life.^18^ The quality of life and overall health of medical practitioners are important in contributing better to the community.

FSD is influenced by various factors, such as sociocultural influences and high educational levels.^7,8^ Marital relationship and characteristics (e.g. partner dissatisfaction, reduced intercourse frequency and number of children), medical conditions (e.g. obesity, diabetes, hypertension and genitourinary problems), hormonal changes (e.g. pregnancy, menopause and breastfeeding) and psychological well-being (e.g. depression, anxiety, sexual abuse history and negative body image) are also important contributing factors.^7,8^ Among medical practitioners, factors such as age, monthly income, work pressure, marital dissatisfaction, frequency of sexual intercourse and poor couple communication are significant contributors to FSD.^11,12,19^ Female medical practitioners have specifically reported a higher prevalence of sexual dysfunction compared to their male counterparts, along with increasing levels of psychological distress.^12^

The impact of FSD is considerable, causing mental and physical distress, impairing family relationships and leading to marital dissatisfaction and divorce.^20-23^ This situation is further complicated for medical practitioners due to the demanding nature of their work environment, which limits personal time and potentially exacerbates mental health challenges.^15,24^ The divorce rate among healthcare professionals is notably high, with medical practitioners in the US experiencing a divorce rate of 24.3% in 2014.^25^ In Malaysia, the divorce rate also increased from 7 to 7.7 per thousand population in 2019.^26^ Based on data from the Department of Shariah Judiciary Malaysia, sexual dissatisfaction is one of the top five main causes of divorce in Malaysia.^27^

In Malaysia, women’s sexual health is a sensitive and culturally complex issue, with stigma around discussing sexual matters.^28^ Conservative cultural norms further hinder open discussions about sexual health, making investigations and interventions challenging.^28^ Screening and diagnosis of FSD at the primary care level are crucial, and intervention is available to improve outcomes. Psychological interventions such as cognitive behavioural therapy and marital counselling can help, while primary care offers treatment for lifestyle and medical factors contributing to FSD. Referrals to specialists such as psychiatrists, gynaecologists or endocrinologists can further address underlying causes.^15,29^ Thus, in view of the increasing prevalence of FSD, the significance of the condition and the scarcity of literature regarding FSD among PCPs, this study aimed to assess the prevalence and associated factors of FSD among PCPs.

## Methods

### Study design and population

A cross-sectional study was conducted among PCPs in Malaysia using a self-administered online questionnaire. The data collection period was 6 months, spanning from August 2023 to January 2024. The participants recruited were female PCPs (including medical officers and family medicine specialists practising within the last 6 months) who were sexually active (defined as at least one sexual activity within the last 6 months) and had consented to the research. Those who were pregnant, within 2 months postpartum or diagnosed with psychiatric illnesses were excluded from the study.

### Sample size calculation

Several sample sizes were calculated in accordance with the study objectives, using OpenEpi version 3.01. Calculations were performed using the formula for ‘sample size for proportion or descriptive study’. The sample size was determined from the largest sample size calculated, using the prevalence of 49.7% as reported by Li et al., based on a significance level of α=0.05, a power of 80% and a confidence interval (CI) of 95%, resulting in a minimum sample size of 382 required.^12^ Given a 40% non-response rate for the online questionnaire, the aim was to distribute to at least 534 participants.^30^ The sample size calculation was based on the single-proportion formula:

Sample size,

n=[DEFF X Np(1-p)/[d^2^/Z^2^_1-∝/2x_(N-1)+p(1-p)]

n = Sample size

DEFF = Design effect (for cluster service) = 1

N = Population size (for finite population factor) = 1,000,000

p = Hypothesised % frequency of outcome factor in the population=0.566

Z^2^_1-∝/2X_ = CI=1.96

d = Desired precision = 5%

### Sampling method, data collection and ethical approval

The three largest organisations and network for PCPs namely the Family Medicine Specialists Association, the Academy of Family Physicians Malaysia and the Malaysia Primary Care Network were approached using purposive and convenience sampling until the target sample size was achieved. The background and objectives of the study were explained to the committee, and with their agreement, the invitation link for the study was shared with their organisation members across the country via email, WhatsApp, Telegram or social media messages. A reminder was sent after 4- and 8-week intervals from the initial invitation. Those who clicked on the link were redirected to the online Google Form.

The Google Form consisted of subsections that started by explaining the background, purpose and benefits of the study. This was followed by checking participation details to ensure they fit the inclusion and exclusion criteria. Individuals who fulfilled the criteria proceeded to the explanation of the study procedure, and the researcher’s contact information was provided. Participants were required to complete an informed consent sheet before proceeding to the study tool to answer the questionnaire voluntarily. The questionnaire ensured participant anonymity; the forms were set to avoid collecting email addresses; and the response was set limited to one per email. An option to provide an email address was given if participants wanted feedback on their responses or needed to be contacted regarding issues such as severe depression, anxiety, stress or FSD. At the end of the form, they were provided with a YouTube link on further information regarding FSD, stress, depression and breathing relaxation techniques. Ethical approval was obtained from the Research Ethics Committee of Universiti Teknologi MARA (reference no. 100-FPR, PT.9/19) (FREC-05-23-02).

### Study tools

The self-administered online questionnaire was divided into three sections. Section A included 21 items that gathered data on sociodemographic, marital and clinical characteristics. Participants filled in or selected options for sociodemographic details such as age, ethnicity, religion, marital status, educational attainment and household income. Marital characteristics included marital duration, number of children, partner absence, frequency of sexual intercourse and contraception methods. Clinical characteristics focused on body mass index, smoking status, diabetes mellitus, hypertension, genitourinary issues, menopause, circumcision history and current breastfeeding status.

Section B comprised 19 items assessing sexual function across six domains, using the Malay Version of the Female Sexual Function Index (MVFSFI) by Sidi et al.^31^ This questionnaire has been adapted and validated for local use from the original FSFI by Rosen et al., with a Pearson correlation coefficient of 0.70–0.973 and a Cronbach’s alpha of 0.87–0.97.^32^ It underwent a validity study and uses a response scale of 5 or 6 points, where higher scores indicate better sexual function. Their study found that the clinical diagnosis based on the DSM-IV and the MVFSFI was comparable, with a sensitivity of 98.8% and a specificity of 98.5%. A total score of ≤55 out of 95 suggests a higher likelihood of FSD (sensitivity: 99%, specificity: 97%). The questionnaire is divided into six domains: desire disorder (items 1 and 2; cutoff score ≤5), arousal disorder (items 3–6; cutoff score ≤9), lubrication disorder (items 7–10; cutoff score ≤10), orgasm disorder (items 11–13; cutoff score ≤4), satisfaction disorder (items 14–16; cutoff score ≤11) and pain disorder (items 17–19; cutoff score ≤7).^31,32^ The total score may detect FSD even without specific domain disorders, or there may also be overlapping disorders. Permission from the original author was obtained for this study.

Section C comprised the Malay version of the Depression, Anxiety and Stress Scale (DASS-21), a self-reported tool used to screen for these conditions. This tool was translated and validated by Ramli et al. in 2007, with a Cronbach’s alpha of 0.84 for depression, 0.74 for anxiety and 0.79 for stress, with interscale correlation coefficients ranging from 0.54 to 0.68. Each of the three scales contains seven items scored on a 4-point Likert scale. Scores are summed, and cutoff points indicate severity levels from normal to extremely severe. Severity levels based on the scores are categorised as follows: For the depression scale, a score of 0-9 indicates normal; 10–13, mild; 14–20, moderate; 21–27, severe; and ≥28, extremely severe. For the anxiety scale, a score of 0–7 indicates normal; 8–9, mild; 10–14, moderate; 15–19, severe; and ≥20, extremely severe. Finally, for the stress scale, a score of 0–14 indicates normal; 15–18, mild; 19–25, moderate; 26–33, severe; and ≥34, extremely severe. The DASS-21 is available for public use, so permission is not required.^33^

### Operational variable definition

This study’s dependent variable, FSD, was operationally defined as an MVFSFI score of ≤55, indicating an inability to experience sexual pleasure, while a score above 55 indicated the absence of FSD.^2,31^ Educational attainment was categorised into degree holders, postgraduate trainees (pursuing further specialist degrees) and postgraduate degree holders (either Master’s or PhD). Household income was categorised into the bottom 40% (B40) (≤RM 6338), middle 40% (M40) (RM 6339 to RM 10,959) and top 20% (T20) (≥RM 10,960) income.^34^ Working hours per week were classified as either normal (≤45 hours) or long (>45 hours).^35^ Clinical characteristics included any history of genitourinary problems (e.g. urinary incontinence, uterine collapse, recurrent urinary tract infection or pelvic inflammatory disease)^36^ and history of female circumcision (traditional practice that involves injury, pricking or nicking of the tip of the clitoris skin).^37^ Psychological well-being was screened using the DASS-21, whereby scores of >10 indicate depression; ≥8, anxiety; and ≥15, stress.^33^

### Statistical analysis

Data entry and statistical analysis were conducted using IBM SPSS Statistics for macOS, Version 29.0 (IBM Corp., Armonk, NY, USA). Descriptive statistics were used to describe the sociodemographic, marital and clinical characteristics; psychological wellbeing; and FSD. Normality was checked using histogram plots. Categorical data were presented as frequencies and percentages.

Chi-square tests were performed to compare categorical variables, with P<0.05 indicating significant differences between groups. Inferential analysis was used to assess the association between the sociodemographic, marital and clinical characteristics; psychological well-being; and FSD among PCPs. Univariate binary logistic regression was utilised to estimate crude odds ratios and 95% CIs. Variables with P-values of <0.25 were then included in a multivariate binary logistic regression to adjust for confounding factors and estimate adjusted odds ratios (aORs). The significance level was set at P<0.05. Nagelkerke R^2^, model fitness, interactions between variables, multicollinearity, ROC curve and Cook’s influential statistics were checked.

## Results

### Prevalence of FSD

The overall prevalence of FSD among the PCPs was 14.7% (95% CI: 11.1, 18.2). The most affected domain was desire disorder, which had a prevalence of 25.4% (95% CI: 21.06, 29.72). The prevalence was 12% (95% CI: 8.87, 15.21) for satisfaction disorder, 11.8% (95% CI: 8.64, 14.92) for orgasm disorder and pain disorder and 11.5% (95% CI: 8.4, 14.64) for lubrication disorder. Arousal disorder had the lowest prevalence at 10.5% (95% CI: 7.49, 13.45) (Table 1). Within the subdomains of FSD, there were several cases that fulfilled the individual cutoff point for the specific disorder but did not fulfil the scoring for FSD.

**Table 1:** Prevalence and subdomains of female sexual dysfunction (N=382).

Parameter		n	% (95% CI)
Female sexual dysfunction		56	14.7 (11.1, 18.2)
Female sexual dysfunction subdomains	Arousal disorder	40	10.5 (7.49, 13.45)
	Orgasm disorder	45	11.8 (8.64, 14.92)
	Pain disorder	45	11.8 (8.64, 14.92)
	Desire disorder	97	25.4 (21.06, 29.72)
	Satisfaction disorder	46	12.0 (8.87, 15.21)
	Lubrication disorder	44	11.5 (8.4, 14.64)

CI: confidence interval

### Demographic characteristics

A total of 4000 PCPs were approached, with 553 responses to the questionnaire resulting in a response rate of 13.8%. However, only 382 were eligible and completed the questionnaire. Most participants and their partners were aged 30-39 years, with partners being in the same age group (48.7%). The majority were married (94.8%) and of Bumiputera ethnicity (81.9%). More than half (55.8%) were degree holders.

In terms of marital and clinical characteristics, the median duration of marriage was 8 years, with 85.3% being married for less than 8 years. Most participants had two or more children (61.5%). Two-thirds of the PCPs (76.2%) worked ≤45 hours a week. The majority (98.4%) had not yet reached menopause. Regarding sexual activity, 58.1% reported having sexual intercourse once or more times per week. In terms of psychological well-being, 16% screened positive for depression, 18.1% for anxiety and 13.1% for stress. In comparison, among the participants with FSD, the majority were also married (85.7%) and of Bumiputera ethnicity (75%). More than half (69.6%) had degree-level education (69.6%) compared to higher educational attainment, and the majority (73.2%) reported reduced sexual intercourse of two or fewer times per month.

Based on the cross-tabulation between FSD and non-FSD with the sociodemographic, marital, clinical and psychological well-being characteristics, there were significant differences found in ethnicity, marital status, educational attainment, frequency of sexual intercourse, depression, anxiety and stress with the presence of FSD (Table 2).

**Table 2:** Sociodemographic, marital, clinical and psychological well-being characteristics among the primary care practitioners (N=382).

Variable		Frequency, n (%)			X^2^ (df)	P-value
		Total (N=382)	FSD (n=56)	No FSD (n=326)		
Age (year) (DOSM)	<30	38 (9.9)	6 (10.7)	32 (9.8)	0.163 (2)	0.922
	30–39	301 (78.8)	43 (76.8)	258 (79.1)		
	≥40	43 (11.1)	7 (12.5)	36 (11)		
Ethnicity	Bumiputera	313 (81.9)	42 (75)	271 (83.1)	6.088 (2)	0.048^*^
	Chinese	29 (7.6)	26 (46.4)	3 (0.9)		
	Indian	40 (10.5)	11 (19.6)	29 (8.9)		
Religion	Islam	312 (81.7)	49 (87.5)	263 (80.6)	4.308 (3)	0.23
	Christian	22 (5.8)	5 (8.9)	17 (5.2)		
	Buddha	18 (4.7)	3 (5.4)	15 (4.6)		
	Hindu	30 (7.8)	5 (8.9)	25 (7.7)		
Marital status	Single/widowed/divorced	20 (5.2)	8 (14.3)	12 (3.7)	10.833 (1)	^1^0.004^*^
	Married	362 (94.8)	48 (85.7)	314 (96.3)		
Partner’s age (year) (DOSM)	<30	38 (9.9)	7 (12.5)	31 (9.5)	0.515 (2)	0.773
	30–39	263 (68.8)	38 (67.9)	225 (69)		
	≥40	81 (21.2)	11 (19.6)	70 (21.5)		
Age difference between partners	Younger partner	120 (32.1)	17 (30.4)	103 (31.6)	1.742 (2)	0.419
	Same age	186 (48.7)	30 (53.6)	156 (47.9)		
	Older partner	76 (19.9)	9 (16.1)	67 (20.5)		
Educational attainment	Degree	213 (55.8)	39 (69.6)	174 (53.4)	5.127 (1)	0.024^*^
	Postgraduate/Master’s/PhD	169 (44.2)	9 (30.4)	160 (46.6)		
Household income/month (RM)	B40 (<6338)	26 (6.8)	5 (8.9)	21 (6.4)	0.769 (2)	0.526
	M40 (6339–10,959)	141 (36.8)	30 (53.6)	111 (34.0)		
	T20 (≥210,960)	215 (56.3)	21 (37.5)	194 (59.6)		
Working hours/week (Employment Act 1995)	≤45	291 (76.2)	38 (67.9)	253 (77.6)	0.2504 (1)	0.114
	>45	91 (23.8)	18 (32.1)	73 (22.4)		
Marital characteristics	Marital duration (year)	<8	178 (46.6)	31 (55.4)	2.042 (1)	0.155
	≥8	204 (53.4)	25 (44.6)			
Number of children	Nulliparous	58 (15.2)	12 (21.4)	46 (14.1)	1.982 (1)	0.159
	≥1	324 (84.8)	44 (78.6)	280 (85.9)		
Staying with partner	Yes	311 (81.4)	42 (75)	269 (82.5)	1.784 (1)	0.182
	No	71 (18.6)	14 (25)	57 (17.5)		
Frequency of sexual intercourse	≤ 2 times/month	160 (41.9)	41 (73.2)	119 (36.5)	26.460 (1)	<0.001^*^
	≥ 1 time/week	222 (58.1)	15 (26.8)	207 (63.5)		
Contraception	No	101 (26.4)	15 (26.8)	86 (26.4)	0.11 (2)	0.994
	Long term	108 (28.3)	16 (28.6)	92 (28.2)		
	Short term	173 (45.3)	25 (44.6)	148 (45.4)		
Clinical characteristics						
Smoking status	No	379 (99.2)	55 (98.2)	324 (99.4)	0.843 (1)	0.379
	Yes	3 (0.8)	1 (1.8)	2 (0.6)		
Obesity (BMI of ≥27.5 kg/m²)	No	282 (74.2)	40 (71.4)	242 (74.7)	0.266 (1)	0.606
	Yes	98 (25.8)	16 (28.6)	82 (25.3)		
Diabetes mellitus	No	376 (98.4)	54 (96.4)	322 (98.8)	1.699 (1)	^1^0.215
	Yes	6 (1.6)	2 (3.6)	4 (1.2)		
Hypertension	No	366 (95.8)	53 (94.6)	313 (96)	0.233 (1)	^1^0.715
	Yes	16 (4.2)	3 (5.4)	13 (4)		
Genitourinary problem	No	373 (97.6)	55 (98.2)	318 (97.5)	0.93 (1)	^1^1
	Yes	9 (2.4)	1 (1.8)	8 (2.5)		
Menopause	No	376 (98.4)	53 (94.6)	323 (99.1)	6.085 (1)	^1^0.43
	Yes	6 (1.6)	3 (5.4)	3 (0.9)		
Circumcision history	No	192 (50.3)	30 (53.6)	162 (49.7)	0.288 (1)	0.592
	Yes	190 (49.7)	26 (46.4)	164 (50.3)		
Breastfeeding	No	314 (82.2)	45 (80.4)	269 (82.5)	0.152 (1)	0.697
	Yes	68 (17.8)	11 (19.6)	57 (17.5)		
Psychological well-being (DASS-21)						
Depression	No (<10)	321 (84)	38 (67.9)	283 (86.8)	12.793 (1)	<0.001^*^
	Yes (≥10)	61 (16)	18 (32.1)	43 (13.2)		
Anxiety	No (<8)	313 (81.9)	40 (71.4)	273 (83.7)	4.896 (1)	0.027^*^
	Yes (≥8)	69 (18.1)	16 (28.6)	53 (16.3)		
Stress	No (<15)	332 (86.9)	42 (75)	290 (89)	8.184 (1)	0.004^*^
	Yes (≥15)	50 (13.1)	14 (25)	36 (11)		

* Significant differences, n (%): sample size for categorical variables, amean (standard deviation) and bmedian (interquartile range) for continuous variables

1 Fisher's exact test was used.

RM: Ringgit Malaysia (currency), SI: sexual intercourse, BMI: body mass index, FSD: female sexual dysfunction, DASS 21: Depression, Anxiety and Stress Scale, DOSM: Department of Statistic Malaysia

### Factors associated with FSD

The univariate logistic regression identified having an Indian ethnicity, having a Hindu religion, being unmarried, having an older partner, attaining postgraduate degree, working ≥45 hours per week, having a marital duration of ≥8 years, not staying with a partner, having sexual intercourse two or fewer times per month, not having diabetes mellitus, not being in menopause and experiencing stress, anxiety or depression were potential associated factors of FSD (P<0.25) (Table 3).

All 14 variables (P<0.25) were analysed and included in the multivariate logistic regression, with the backward stepwise model identifying three independent factors of FSD. The participants who had sexual activity two or fewer times per month had 4.88 times higher odds of developing FSD (aOR: 4.88; 95% CI: 2.522, 9.442; P<0.001). Those with depression had 3.45 times higher odds of developing FSD (aOR: 3.45; 95% CI: 1.653, 7.204; P<0.001). Finally, the participants who attained basic degrees had 2.66 times higher odds of developing FSD (aOR: 2.66; 95% CI: 1.338, 5.285; P=0.005), further emphasising the complexity of the factors influencing FSD among this population. The factors associated with FSD are shown in Table 4.

**Table 3:** Univariate analysis of the factors associated with FSD (n=56).

Variable		Wald statistic^a^	Crude OR (95% CI)^a^	(<0.25)^a^
Age (year) (DOSM)	<30	1.620	1	0.922
	30-39	0.062	0.889 (0.351, 2.252)	0.804
	≥40	0.004	1.037 (0.316, 3.408)	0.952
Ethnicity	Bumiputera	5.767	1	0.056^*^
	Chinese	0.218	0.745 (0.216, 2.569)	0.641
	Indian	5.240	2.447 (1.137, 5.267)	0.022^*^
Religion	Islam	4.131	1	0.248^*^
	Christian	0.443	1.469 (0.473, 4.557)	0.506
	Buddha	0.182	1.322 (0.367, 4.766)	0.670
	Hindu	3.874	2.404 (1.004, 5.756)	0.049^*^
Marital status	Single/widowed/divorced	9.335	1	
	Married	0.789	0.229 (0.089, 0.59)	<0.002^*^
Partner’s age (DOSM)	<30	0.511	1	0.774
	30-39	0.410	0.748 (0.307, 1.820)	0.522
	≥40	0.469	0.59 (0.247, 1.964)	0.494
Age difference	Younger partner	1.724	1	0.422
	Same age	0.703	1.342 (0.675, 2.667)	0.402
	Older partner	1.708	1.710 (0.765, 3.822)	0.191^*^
Educational attainment	Degree	4.993	2.004 (1.089, 3.687)	0.025^*^
	Postgraduate/Master’s/PhD		1	
Household income/month (RM)	B40 (<6338)	0.522	1	0.77
	M40 (6339-10,959)	0.312	0.735 (0.25, 2.164)	0.576
	T20 (≥10,960)	0.515	0.681 (0.239, 1.944)	0.473
Working hours/week (Employment Act 1995)	≤45		1	
	>45	0.2469	1.642 (0.885, 3.047)	0.116^*^
Marital duration (year)	<8		1	
	≥8	1.006	0.662 (0.374, 1.171)	0.157^*^
Staying with partner	Yes		1	
	No	1.762	1.573 (0.806, 3.071)	0.184^*^
Number of children	Nulliparous		1	
	≥1	1.969	0.602 (0.296, 1.226)	0.162
Frequency of sexual intercourse	≤2 times/month	1.559	4.755 (2.525, 8.954)	<0.001^*^
	≥1 time/week		1	
Contraception	No	0.11	1	0.994
	Long term	0.00	0.994 (0.465, 2.139)	0.994
	Short term	0.008	0.968 (0.484, 1.937)	0.928
Smoking status	No		1	
	Yes	0.767	2.945 (0.263, 33.04)	0.381
Obesity (BMI of ≥27.5 kg/m^2^)	No		1	
	Yes	0.067	1.176 (0.625, 2.211)	0.616
Diabetes mellitus	No		1	
	Yes	1.547	0.335 (0.06, 1.876)	0.214^*^
Hypertension	No		1	
	Yes	0.222	0.638 (0.202, 2.662)	0.638
Genitourinary problem	No		1	
	Yes	0.092	1.384 (0.17, 11.282)	0.762
Menopause	No		1	
	Yes	4.744	0.164 (0.032, 0.835)	0.029^*^
Circumcision history	No		1	
	Yes	0.287	1.168 (0.662, 2.062)	0.592
Breastfeeding	No		1	
	Yes	0.152	2.060 (1.075, 3.947)	0.697
DASS 21 – Anxiety	No		1	
	Yes	4.749	3.118 (1.634, 5.948)	0.029^*^
DASS 21 – Depression	No		1	
	Yes	11.898	3.118 (1.634, 5.948)	<0.001^*^
DASS 21 – Stress	No		1	
	Yes	7.715	2.685 (1.337, 5.391)	0.005^*^

a Univariate logistic regression

* P-value included in a backward stepwise multivariate logistic regression model

OR: odds ratio, CI: confidence interval, FSD: female sexual dysfunction, BMI: body mass index, B40: bottom 40% (<6338), M40: middle 40% (6339-10,959), T20: top 20% (>10,960), DASS-21: Depression, Anxiety and Stress Scale, DOSM: Department of Statistic Malaysia

**Table 4:** Multivariate analysis of the factors associated with FSD (n=56).

Variable		Wald statistic^b^	Crude OR (95% CI)	P-value (<0.25)^b^
Ethnicity	Bumiputera	6.162	1	0.056
	Chinese	2.059	0.379 (0.101, 1.426)	0.151
	Indian	3-324	2.253 (0.941,5.393)	0.068
Religion	Islam	2.255	1	0.542
	Christian	0.508	3.042 (0.28, 33.0310)	0.360
	Buddha	0.635	0.388 (0.90, 1.573)	0.204
	Flindu	0.081	0.998 (0.60, 16.702)	0.999
Marital status	Single/widowed/divorced		1	
	Married	1.022	0.450 (0.096, 2.114)	0.312
Age difference	Younger partner	0.833	1	0.659
	Same age	0.725	1.402 (0.644, 3-014)	0.394
	Older partner	0.025	1.078 (0.420, 2.768)	0.875
Educational attainment	Degree	7.787	2.659 (1-338, 5.285)	0.005^*^
	Postgraduate/Master’s/PhD		1	
Working hours/week (Employment Act 1995)	≤45		1	
	>45	0.476	0.749 (0.33,1.70)	0.490
Marital duration (year)	<8		1	
	≥8	1.036	0.692 (0.341, 1.406)	0.309
Staving with partner	Yes		1	
	No	1.469	0.552 (0.221, 1.444)	0.226
Number of children	Nulliparous		1	
	≥1	0.393	1.446 (0.456, 4.581)	0.531
Frequency of sexual intercourse	≤2 times/month		4.880 (2.522, 9.442)	<0.001^*^
	≥1 time/week	22.15	1	
Diabetes mellitus	No		1	
	Yes	0.319	1.842 (0.221, 15-255)	0.572
Menopause	No		1	
	Yes	2.403	4.687 (0.665, 33.048)	0.121
DASS 21 - Depression	No		1	
	Yes	11.58	3.450(1.653, 7.204)	<0.001^*^
DASS 21 - Anxiety	No		1	
	Yes	0.082	1.156 (0.427, 3.131)	0.775
DASS 21 - Stress	No		1	
	Yes	0.000	0.996 (0.321,3.094)	0.994

b Multivariate logistic regression

* P-value: significant

OR: odds ratio, CI: confidence interval, FSD: female sexual dysfunction, BMI: body mass index, B40: bottom 40% (<6338), M40: middle 40% (6339–10,959), T20: top 20% (>10,960), DASS-21: Depression, Anxiety and Stress Scale, DOSM: Department of Statistic Malaysia

The regression analysis using binary logistic regression and the enter-stepwise method revealed a Nagelkerke R^2^ of 20%, indicating that the independent variables accounted for 20% of the variability in the likelihood of the outcome. The Hosmer-Lemeshow goodness-of-fit test (P=0.402) confirmed that the logistic regression model was a good fit for the data. The twoway interaction analysis among the variables revealed no significant interactions, indicating that the associations observed were independent of one another. The multicollinearity analysis indicated that all variables had acceptable variance inflation factor values (<10), confirming no multicollinearity or highly correlated independent variables. The model demonstrated good discriminatory ability, correctly identifying 75.2% (95% CI: 68.8, 81.7) of cases predicted to be FSD, as evidenced by the area under the ROC curve. Cook’s distance analysis was conducted to identify influential outliers, which confirmed that none were present, ensuring the reliability and stability of the model.

## Discussion

This study determined the prevalence and associated factors of FSD among PCPs in Malaysia. The results showed a prevalence of 14.7% (95% CI: 11.1,18.2), which is notably higher than the previously reported rate of 5.5% among healthcare providers in Malaysian hospitals, as noted by Grewal et al. in 2013.^13^

The key difference is our study focused on the primary care setting involving female PCPs in Malaysia, whereas Grewal et al. conducted their research across three hospitals, encompassing all healthcare personnel, including 32 medical practitioners, nurses, hospital attendants and allied health staff. Thus, our study reflects a higher prevalence possibly due to its focus on primary care doctors with high job demands. In comparison to previous studies on FSD prevalence among female medical practitioners globally, a prevalence of 49.7% and 40% was reported by Li et al. in 2020^12^ and Fekih-Romdhane et al. in 2019.^11^ The lower prevalence identified in our study may be attributed to the different study tools and the specific populations involved; for instance, Li et al. utilised the ASEX in a multicentre hospital setting focused on female practitioners with high job stress levels and poor quality of life, while Fekih-Romdhane et al. used the Arabic version of the FSFI but focused on a smaller sample size of married female practitioners in a military hospital, which was highly associated with depression, anxiety and stress. The lower prevalence may also be due to several factors inherent in the Malaysian sociocultural landscape, which likely contributed to significant underreporting and underdiagnosis of FSD. The nation’s conservative cultural beliefs and norms create a pervasive reluctance among PCPs to openly discuss sexual health issues, limiting both disclosure and proactive screening.^28^ The study sample was predominantly Malay Muslims, as discussions about sexuality remain taboo and are often deemed unimportant within this population.^28^

Our study’s findings showed that the desire domain of FSD was the most significantly affected, with a prevalence of 25.4% (95% CI: 21.06, 29.72). This aligns with the findings of the local study by Grewal et al. (18.9%). In contrast, Tey et al. reported a higher prevalence of 85.2%, possibly due to their demography of middle-aged women with medical comorbidities in an outpatient hospital clinic.^10,38^ Most women hold back from sharing their sexual desires with their partners in view of sociocultural beliefs, which may contribute to this result.^10^

We found a relatively lower prevalence of other FSD domains, such as satisfaction (12%), orgasm (11.8%), pain (11.8%), lubrication (11.5%) and arousal (10.5%). However, this contrasts with the systematic review by Alidost et al., which reported a higher prevalence of the domains: desire (50.7%), arousal (48.2%), orgasm (40.1%), pain (39%), lubrication (37.6%) and satisfaction (35%),^7^ although the population in their study differed significantly in demographics, lifestyles and clinical characteristics, including a broader range of participants compared to our study. This may reflect the unique stressors and lifestyle demands faced by PCPs and suggests a distinct pattern of sexual difficulties among the participants (i.e. desire and satisfaction) as compared to other population.

In terms of potential factors associated with FSD, among the variables of interest for PCPs was working hours, as medical practitioners frequently experience issues with work burden, which often results in a lack of time spent with their spouses.^39^ Studies have also shown that tiredness results in diminished mental alertness, irritability and a decline in desire for sex.^24^ However, in our study, the multivariate logistic regression showed that this factor was not significantly associated with FSD.

In the multivariate logistic regression analysis, we identified three significant factors of FSD among the PCPs. Lower frequency of sexual intercourse emerged as a significant associated factor, with the individuals having sexual intercourse two or fewer times per month having 4.88 times higher odds of experiencing FSD (aOR: 4.88; 95% CI: 2.552, 9.442; P<0.001) compared to having sexual intercourse one or more times per week. This corresponds with the previous report by Ferguson et al. among medical and surgical residents using a similar study tool, where the results showed that a higher intercourse frequency was significantly associated with being in a relationship and having higher sexual satisfaction.^19^ The association between less frequent sexual activity and increased FSD risk is unsurprising but highlights the potential for a vicious cycle: FSD can lead to decreased sexual activity, which can consequently exacerbate FSD symptoms.

There was also a strong association between positive screening for depression and FSD. The participants with depression had 3.45 times higher odds of experiencing FSD (aOR: 3.45; 95% CI: 1.653, 7.204; P<0.01) than those without depression. Similarly, Fekih-Romdhane et al. reported the presence of depression, anxiety and stress among female medical practitioners with FSD.^11^ Depression may manifest as reduced sexual desire and libido, arousal difficulties and overall dissatisfaction, potentially creating a negative feedback loop that leads to diminished sexual interest and activity.^24^,^39^

This study found that the participants with a basic degree had 2.66 times higher odds of experiencing FSD than the postgraduate trainees or those with Master’s or PhD degrees (aOR: 2.66; 95% CI: 1.338, 5.285; P=0.005). In contrast, Grewal et al. found that a higher educational attainment (degree and postgraduate) was associated with FSD.^13,38^ This may be due to the difference in the study population, as our study focused on PCPs, while Grewal et al. included all healthcare personnel as mentioned above.^13^ Most PCPs in our study had only basic degrees as compared to postgraduate degrees. PCPs with higher levels of education may be more proactive due to their ability to navigate sexuality challenges better.^40^ This finding raises questions about the relationship between educational level and sexual health. Thus, further research is essential to better understand the dynamics of how educational level influences the risk of FSD.

### Study strengths and limitations

To the best of our knowledge, this is the first study to determine the prevalence and associated factors of FSD among PCPs in Malaysia. Well-validated tools were also used for data collection. However, the findings of the study may need to be interpreted subject to several limitations. First, this study was limited to PCPs, reducing the generalisability to all medical practitioners. Second, this study utilised an online convenience sampling method. Thus, the findings may be affected by selection bias. To reduce this, we ensured that the study invitations reached as many PCPs as possible and sent reminders. Finally, there may also be response bias since this study relied on selfreported data that included information about the participants’ sexual nature and lifestyle, which can be subjected to stigma and cultural beliefs. However, we disclosed and ensured that the questionnaire was completely anonymous.

### Implications of the research in clinical practice

This study showed a notably higher prevalence of FSD among PCPs compared to a previous study conducted among healthcare providers in Malaysian hospitals. Although the prevalence is lower in our study compared to other studies worldwide, the increasing trend and burden of FSD in Malaysia and other countries worldwide are significant. This highlights the need to create awareness of FSD among PCPs, educating them on the importance of addressing the issue to improve their help-seeking behaviour and ultimately their quality of life. Recognising the associated factors is also important to understand the cultural and social determinants of female sexual health to tailor screening and intervention strategies.

### Recommendations for future research

Future research should focus on all other medical practitioners to better understand the factors contributing to FSD among different demographics. Additionally, further research should explore the knowledge of FSD among PCPs to aid in targeted education and improve health programmes for the detection and management of FSD among both healthcare providers and their patients.

## Conclusion

This study identified a higher prevalence of FSD among PCPs in Malaysia compared to a previous local study on healthcare workers, with the most prevalent issue of desire disorder. There were significant associations between reduced frequency of sexual intercourse, depression and educational attainment and FSD. These findings highlight the multifaceted biopsychosocial concern in FSD among PCPs, illuminating the need for a holistic approach addressing both physical and mental health to enhance their overall well-being. Understanding the cultural determinants of FSD is important to tailor screening and intervention strategies.

## Data sharing statement

The dataset used and/or analysed during this study is available from the corresponding author on reasonable request and subjected to data protection regulations.

**How does this paper make a difference in general practice?**

- This study highlights the rising prevalence of female sexual dysfunction (FSD) among Malaysian primary care practitioners (PCPs) and the need to raise awareness regarding FSD.
- Educating PCPs on FSD is important to improve their help-seeking behaviour and quality of life, enabling them to serve the community effectively.
- Understanding cultural and social determinants relative to FSD helps tailor effective intervention strategies.
- Acknowledging the burden and impact of FSD is crucial for policy development to enhance screening, diagnosis and treatment, aligning with Sustainable Development Goals 3 and 5.

## Appendix

**1. Permission from the original author of the FSFI**

**2. Permission from the original author of the MVFSFI**

## References

[1] WHO. (2014). WHO | Sexual health..

[2] Basson R (2005). Women's sexual dysfunction: revised and expanded definitions.. CMAJ..

[3] WHO. (2010). Developing Sexual Health Programmes. A Framework for Action..

[4] MOH Malaysia. (2008). Saringan Status Kesihatan Lelaki Dewasa (BSSK/L/1/2008)..

[5] MOH Malaysia. (2013). Borang Status Kesihatan Wanita Dewasa (BSSK/W/2008 Pind 1/2013)..

[6] Batliwala Y (2022). Sustainable Development Goal 2030, Goal 5: Gender Equality..

[7] Alidost F, Pakzad R, Dolatian M, Abdi F (2021). Sexual dysfunction among women of reproductive age: A systematic review and meta-analysis.. Int J Reprod Biomed..

[8] McCool-Myers M, Theurich M, Zuelke A, Knuettel H, Apfelbacher C (2018). Predictors of female sexual dysfunction: a systematic review and qualitative analysis through gender inequality paradigms.. BMC Womens Health..

[9] Sidi H, Puteh SEW, Abdullah N, Midin M (2007). The prevalence of sexual dysfunction and potential risk factors that may impair sexual function in Malaysian women.. J Sex Med..

[10] Tey YY, Ching SM, Maharajan MK (2022). Prevalence and factors associated with sexual dysfunction among middle-aged women in a multi-ethnic country: a cross sectional study in Malaysia.. Malays Fam Physician..

[11] Fekih-Romdhane F, Ben Zid A, Ridha R, Masmoudi J, Cheour M (2019). Evaluation of sexual function in a sample of married female resident medical doctors.. Sexologies..

[12] Li W, Li S, Lu P (2020). Sexual dysfunction and health condition in Chinese doctor: prevalence and risk factors.. Sci Rep..

[13] Grewal GS, Gill JS, Sidi H (2014). Prevalence and risk factors of female sexual dysfunction among healthcare personnel in Malaysia.. Compr Psychiatry..

[14] MOH. (2022). Health_Facts_2022-Updated..

[15] Ab Rahman N, Husin M, Dahian K, Mohamad Noh K, Atun R, Sivasampu S (2019). Job satisfaction of public and private primary care physicians in Malaysia: analysis of findings from QUALICO-PC.. Hum Resour Health..

[16] Nelson EG, Henry WF (1978). Psychosocial factors seen as problems by family practice residents and their spouses.. J Fam Pract..

[17] Millman AL, Rebullar K, Millman RD, Krakowsky Y (2021). Female sexual dysfunction - awareness and education among resident physicians.. Urology..

[18] Stamatiou K, Margariti M, Nousi E, Mistrioti D, Lacroix R, Saridi A (2016). Female sexual dysfunction (FSD) in women health care workers.. Mater SocioMedica..

[19] Ferguson GG, Nelson CJ, Brandes SB, Shindel AW (2008). The sexual lives of residents and fellows in graduate medical education programs: a single institution survey.. J Sex Med..

[20] Artune-Ulkumen B, Erkan MM, Pala HG, Bulbul Baytur Y (2014). Sexual dysfunction in Turkish women with dyspareunia and its impact on the quality of life.. Clin Exp Obstet Gynecol..

[21] Daneshfar F, Keramat A (2023). Sexual dysfunction and divorce in Iran: A systematic review.. J Fam Med Prim Care..

[22] Gautam S, Batra L (1996). Sexual behaviour and dysfunction in divorce seeking couples.. Indian J Psychiatry..

[23] Shoji M, Hamatani T, Ishikawa S (2014). Sexual satisfaction of infertile couples assessed using the Golombok-Rust Inventory of Sexual Satisfaction (GRISS).. Sci Rep..

[24] Myers MF (1986). Marital distress among resident physicians.. CMAJ..

[25] Ly DP, Seabury SA, Jena AB (2015). Divorce among physicians and other healthcare professionals in the United States: analysis of census survey data.. BMJ..

[26] Department of Statistics Malaysia. (2019). Press Release Marriage and Divorce Statistics, Malaysia, 2019. Department of Statistics Malaysia..

[27] Lim I (2023). Seven in 10 of Malaysia’s Shariah cases is on divorce; money top cause of Selangor’s Muslims’ marriage breakdown..

[28] Muhamad R, Horey D, Liamputtong P, Low WY, Zulkifli MM, Sidi H (2021). Transcripts of unfulfillment: a study of sexual dysfunction and dissatisfaction among Malay-Muslim women in Malaysia.. Religions..

[29] LPPKN. (2019). Kaunseling Keluarga & Perkahwinan..

[30] Bujang MA (2021). A step-by-step process on sample size determination for medical research.. Malaysian J Med Sci..

[31] Sidi H, Abdullah N, Puteh SE, Midin M (2007). The Female Sexual Function Index (FSFI): validation of the Malay version.. J Sex Med..

[32] Rosen R, Brown C, Heiman J (2000). The Female Sexual Function Index (FSFI): a multidimensional self-report instrument for the assessment of female sexual function.. J Sex Marital Ther..

[33] Ramli M, Salmiah M, Nurul Ain M (2009). Validation and psychometric properties of Bahasa Malaysia version of the Depression Anxiety and Stress Scales (DASS) among diabetic patients.. MJP OnlineEarly..

[34] Department of Statistics Malaysia. (2022). Household Income & Expenditure..

[35] MALAYSIA LO. (2023). Employment Act 1955 (Act 342)..

[36] Biggers A (2023). Understanding genitourinary disorders..

[37] WHO. (2024). Female Genital Mutilation..

[38] Grewal GS, Gill JS, Sidi H, Gurpreet K, Jambunathan ST, Suffee NJ (2013). Sexual desire disorder in female healthcare personnel in Malaysia.. Asia Pac Psychiatry..

[39] Myers MF (2004). Medical marriages and other intimate relationships.. Med JAust..

[40] Younis I, Daifalla A (2023). Academic educational level: influence on female sexual functions..

